# The impact of the Covid-19 related media coverage upon the five major developing markets

**DOI:** 10.1371/journal.pone.0253791

**Published:** 2021-07-01

**Authors:** Zaghum Umar, Mariya Gubareva, Tatiana Sokolova

**Affiliations:** 1 College of Business, Zayed University, Abu Dhabi, UAE; 2 South Ural State University, Chelyabinsk, Russian Federation; 3 ISCAL–Lisbon Accounting and Business School, Instituto Politécnico de Lisboa, Lisbon, Portugal; 4 Centre for Financial Research & Data Analytics, National Research University Higher School of Economics / HSE University, Moscow, Russian Federation; 5 SOCIUS / CSG—Research in Social Sciences and Management, Lisbon, Portugal; 6 National Research University Higher School of Economics / HSE University, Moscow, Russian Federation; The Bucharest University of Economic Studies, ROMANIA

## Abstract

This paper analyses the influence of the Covid-19 coverage by the social media upon the shape of the sovereign yield curves of the five major developing countries, namely Federative Republic of B razil, Russian Federation, Republic of India, People’s Republic of China, and the Republic of South Africa (BRICS). The coherenc e between the level, slope, and the curvature of the sovereign yield term structures and the Covid-19 medi a coverage is found to vary between low and high ranges, depending on the phases of the pandemic. The empirical estimations of the yield-curve factors a re performed by means of the Diebold–Li modified version of the Nelson–Siegel model. The intervals of low coherence reveal the capacity of the two latent factors, level and slope, to be used for creating cross-factor diversification strategies, workable under crisis conditions, as evidenced on the example of the ongoing pandemic. Diverse coherence patterns are reported on a per-country basis, highlighting a promising potential of sovereign debt investments for designing cross-country and cross-factor fixed-income strategies, capable of hedging downside risks.

## 1. Introduction

The ongoing global coronavirus outbreak triggered an unprecedented financial and economic crisis, bringing tremendous uncertainty to all sides of human life [1–4]. Many developing and developed states have drastically restricted people´s mobility aiming to contain further advancement in the virus propagation. Global economy and financial markets have been profoundly impacted by this Covid-19 fueled crisis ([5–12]; among others). The pandemic crisis has brought about unforeseen volatility in government bond yields. The respective yield curves have proved to be highly volatile along the apogee of the unique market meltdown and economic shock in March-April 2020. In this global climate of high uncertainty, it is desirable to reassess the diversification potential and eventual safe haven attribute of the sovereign debt, issued by leading emerging market (EM) countries. Being inspired by [12], dealing with the UST securities´ yield-curve analyses and hedge properties, in this paper we study the shape of the yield curves of the five major emerging EM e conomies, namely, as Federative Republic of Brazil, Russian Federation, Republic of India, People’s Republic of China, and the Republic of South Africa, being jointly known as BRICS.

Specifically, this paper investigates the interrelation between the sovereign yield curves decomposed into the level, slope, and curvature components, and the pandemic media coverage. It contributes to the literature along the three following strands. First, by providing dynamic inter-dependence heatmaps, we complete a current void in scientific investigation of interrelations between the Covid-19 coverage in social media and the shape of the sovereign yield curves of BRICS. As far as we are aware, our research is the pioneer application of the wavelet analysis to address the changes in the shape of the yield curves in the wake of Covid-19 vis-a-vis intensity of the media coverage of the pandemic. Second, we advance the state-of-the-art in respect to the influence of the pandemic on EM countries. Due to the contemporaneous topicality of our study, which deals with the Covid-19 meltdown in March, 2020, and the subsequent recovery, its results are potentially insightful for investment professionals seeking arbitrage opportunities and diversifications attributes as well as for policy makers assessing suitability of yield-curve control policies to promote financial stability. Third, multiple intermittent zones, observed in the time-frequency heatmaps for the latent yield-curve factors and mass media, reveal distinctive degrees of coherence ranging from zero to perfect synchronization. Areas of weak coherence point at diversifying capacity of the three latent yield factors, which may be used for designing hedge strategies, suitable not only for normal market conditions, but also during global crises such as the ongoing pandemic outbreak.

The remaining part of the manuscript possesses the following structural order. Section 2 provides the literature review. Section 3 presents wavelet econometric framework. Section 4 addresses the estimation of the yield curves and introduces the three latent yield factors. Section 5 is dedicated to the dataset description and sample statistics. Section 6 discusses the results a nd their implications. Section 7 conveys concluding remarks.

## 2. Literature review

This section provides the literature overview aimed at highlighting the most relevant research aligned with the scope of our paper. Thus, the survey covers the following five topics: (i) Covid-19 impact on financial markets, (ii) forecasting the term structure of interest rates, (iii) media coverage influence on financial markets, (iv) wavelet technique, and (v) spillover, safe-haven attributes and hedging opportunities across diverse investments.

### 2.1. Covid-19 impact on financial markets

In respect of the state of art relative to academic research on diverse financial and economic consequences of the pandemic, including design and implementation of policies facilitating recovery from the Covid-triggered slowdown, it is worth acknowledging that the respective literature has recently grown with a rapid pace ([13–20]; and references therein). However, a major part of academic publications addressing the reaction of financial markets to the pandemic is mostly focused on the stock market impacts ([3, 5, 8, 9, 21, 22]; among others), commodities ([23–27]; among others), currencies and crypto-currencies [28–31]. However, it is worth noting that the sovereign and corporate debt markets are barely addressed. The literature on fixed-income finance during the ongoing Covid-19 fueled turmoil, although rapidly developing,—see [4, 32–34];—is still rather scant, especially regarding the term-structures of the interest rates around the globe. The dynamics of the sovereign yield curves in the wake of the Covid-19 triggered crisis remains rather not addressed with the only known to us exception, dealing with the term-structure of the yield of the US Treasuries; see [35].

### 2.2. Forecasting the term-structure of interest rates

It is worth mentioning that prior to the pandemic outbreak, the literature on the yield term-structures for both developed and developing economies has experienced a significant expansion; see [36–43] among many others. A number of studies have analyzed the interrelationship between the macro—economic parameters and the shape of the sovereign debt curves in US [35, 36, 41, 44–46], Canada [38, 47]] and in the European Union [37, 39, 48]. Other researchers have focused on forecasting the shapes of the yield curves for BRICS [42].

Recently [49] have studied the relationship between the term structure of interest rates and business activity for G7 economies using a seasonally adjusted datasets, derived from the industrial production index. However, the time span of their analysis is limited by 2019, leaving the pandemic times unaddressed. Authors conclude for positive linkages between the term spreads and business activity for all the G7 countries except Italy. All the yield-curve shaping parameters,—level, slope, and curvature,—are helpful for predicting business activity in France and Germany one year into the future. Their results also suggest that adding supplementary macroeconomic parameters, e.g., changes in the gross domestic product and the three-month rate, makes the relationship between the term spread and business activity stronger. [49] conclude that the latent shaping factors of the term-structure of interest rates could be potentially useful for forecasting business activity and designing policy solutions. However, further research in this field, especially incorporating the most recent yield curve behavior under stressed pandemic conditions, becomes highly desirable and a possibility to fill in this gap has motivated our research, although focused on the BRICS countries. The study of emerging economies fixed income markets is particularly important because of their unique risk-return patterns, as pointed out by [50].

### 2.3. Media coverage influence on financial markets

The planetary scale of coronavirus spreading as well as the socioeconomic effects brought about by severe measures undertaken by many countries to contain the advancements of the virulent disease have highlighted a relevance o f the media coverage of the pandemic. It is especially important to make people accept more easily curfews, lock-downs, and other mobility limitations and be prepared for the economic recovery from the Covid-19 slowdown. Behavioral finance literature provides multiple cases when the mass media tracking is used to gauge both, complex behavior of society and that of financial market ([30, 51–57]; and the references therein). In general terms, all these studies agree that media tracking is a powerful mean allowing for data gathering, focused on less rational factors, e.g., market sentiments of investors´ communities, public mood at the level of a society, among many other behavioral drivers.

In particular, [53], use an extensive dataset on the social media announcements about the companies, listed on Taiwan stock exchange. They find that investors´ trades are influenced by count and by quality of the media publications. Whereas distinct groups of investment professionals exhibit varying reactions to the mood of news coverage, the observed trading patterns of foreign or external in vestors are usually consonant with positivism or negativism of media announcements. In line with this research, [55], figure out that companies with larger news count during the preceding month exhibit more appealing sustained stock returns, in comparison to the firms with smaller numbers of publications, during the months to come. They conclude that the effects of publications´ count on stock returns are more pronounced and more positive while in the capital market places, there is a dominance of retail/amateur investing agents. [55], demonstrate that the higher is the level of news coverage, the more sustained is the attention of investment community to a chosen firm. Such attention promotes a push to purchase the stock shares, which forces the price to increase and, hence, results in superior gains from shareholding. However, they recognize that the publications activity affects stock returns differently being dependent upon the traits of investment communities.

Our current research represents continuation of the efforts undertaken in the cited above studies regarding the media coverage influence on financial markets. We believe that encompassing the ongoing Covid-19 period our study is a valuable and relevant contribution towards better understanding of market sentiment and its role in changing shapes of the yield curves of the BRICS countries.

### 2.4. Time-frequency wavelet analysis

Amidst diverse approaches employed in the field of econometrics to study the interrelations of investors´ sentiments and dynamics of capital markets, we choose the wave let–based approach allowing for analyses in the time—frequency space. We would like to mention that this technique has been already employed in this area of research for studying the interrelations of investors´ sentiments in the developed and developing markets as in [58]. Notwithstanding, authors do not employ primary information regarding the news publications metrics, using instead a set of chosen sent iment indices, which represent pre-treated data series.

Following this strand of literature, we build our investigation on several previous studies based on wavelet analysis [30, 36, 57, 59–61]. The time-frequency wavelet-based analysis is employed herein for studying the influence of the pandemic-related news coverage upon the shapes of the governmental yield term structures of the BRICS. The model, originally developed in [62] for studying the dynamic nature of the yield curves, is used in our research in its advanced specification, as in [63]. This methodology approximates the entire term structure by means of the three most relevant yield-curve shaping parameters, being them the level, slope, and the curvature factors; see [64].

The wavelet-based framework allows obtaining results in a format of heatmaps in the time–frequency space, which contain information, simultaneously, on pair-wise co herence and time-difference of the considered pairs of variables. Because of this peculiarity, such method of analysis facilitates a joint consideration of information coming from the frequency and time domains. The wave let transformation is widely applied in many areas of scientific research. For instance [65], apply the wavelet transformation approach for estimating river sediments using data decomposition relative to each distinct phase of sediment load. In parallel [66], apply a wavelet–coupled random vector functional link network to forecast coronavirus spreading in the five worst-affected states, namely, Federative Republic of Brazil, Republic of India, Republic of Peru, Russian federation, and the United States of America. It is also worth mentioning few recent applications of wavelet technique to study the pandemic impacts upon capital markets: [30, 33, 60, 67, 68], and the references therein.

We acknowledge an existence of a wide range of diverse econometric techniques applicable for studying coherence and contagion patterns, namely, VaR ([69], among many others), entropy-based fuzzy least squares twin support vector machine approach [12], several variance decomposition and time–varying connectedness approaches ([70], and the references therein), unconstrained convex minimization based implicit Lagrangian twin extreme learning machine technique [71], density-weighted support vector machines approach [72], among many other techniques, we stay with the wavelet-based approach because of the rationale discussed below.

As the first argument, we highlight that the wavelet coherence technique is an appropriate instrumentarium for obtaining valuable knowledge regarding a simultaneous dynamics of a pair of data-series, as its outputs provide information in both, time and frequency dimensions. It turns to be especially suitable for studying lead-lag patterns and co-movements between indices. Taking into consideration a relevance of considering distinct investment time scales, i.e., frequency ranges, in the context of our research, our natural choice stays with the wave let methodologies. The second point is that for the wavelet method to be employed no hardly restricting assumptions, e.g., station arity of processes, are needed. Hence, this technique is appropriate to investigate both non-linear and linear phenomena. The third attractive feature of the followed by us approach is related to the capacity of the wave let methodology to provide relevant findings, especially so in reference to time-limited data-series, as in the case of the relatively short Covid-19-concerned time-spans of data. Wrapping-up, the above addressed characteristics of the wave let technique certify it as a robust scientific method, widely used to study coherence patterns brought about by jointly analyzed diverse arrays of data [33, 59, 60, 73]. Herein, we follow the wavelet-based approach to investigate lead and lag interrelations of the variations in levels of the pandemic media coverage and shaped of the yield curves of five major developing countries.

In particular, amidst previous works, we would highlight the research by [33] focused on a time-frequency analysis of spreads and total returns dynamics of emerging market debt during the initial phase of the Covid-19 pandemic. This study proves the capacity of the wavelet-based approach to be a powerful tool in producing valuable knowledge on interdependencies in the dynamics of the Covid-19 media coverage index and important parameters describing emerging markets debt, simultaneously analyzing them in a time-frequency space. This potentially enables us to study various patterns of the interrelationship between the media coverage and the yield-curve parameters. Further on, the phase-difference tech nique is used in our research to generate a valuable knowledge regarding the direction of the comovements and, hence, to study causal interrelations of the variations in the level of the pandemic news coverage and changes in the term structure of the sovereign interest rates of the BRICS.

### 2.5. Spillovers, safe-haven attributes and hedging opportunities across diverse investments

Overall, the literature on spillovers, safe-haven attributes and hedging opportunities across diverse investments, including governmental debt started, especially since the Global Financial Crisis of 2007/2008, to attract a strong interest of academic community; see [35, 74–80]. The ongoing global coronavirus outbreak represents an exceptional research opportunity and drives an important strand of scientific works, dedicated to the influence of the Covid-19 circumstances on global capital markets. In a wake of the above mentioned works, we contribute to this strand of scientific research by documenting the responses of the sovereign yield curves of the BRICS to the pandemic-triggered meltdown and the initial recovery from it. Our results are potentially useful for investment professional searching for arbitrage opportunities along an individual sovereign curve or pursuing diversification with trades between similar or distinct maturity points of different yield curves. It is especially so as the uncommon yield-curve dynamics along the pandemics is addressed.

## 3. Wavelet econometric framework

We apply the squared wavelet coherence (SWC) econometric framework along with the wave let coher ence phase-difference (WCPD) analysis in line with [81, 82]; as well as following contemporary research [29, 30]. We analyze the estimated daily time series, describing the level, scope and curvature yield factors, considered jointly with the daily data series of the pandemic media coverage index (MCI). The estimation of the yield curves will be addressed in the next Section 4. The time interval of our research covers the first ten months of the pandemic year 2020: from January 01 to October 26. The interrelations of the Covid-19 coverage by the news providers, gauged with the help of the MCI, and the latent sovereign yield factors for the BRICS are analyzed.

We analyze the interrelations of the MCI and the yield-curve shaping parameters of level, slope, and curvature employing the SWC analysis. Continuous wave let trans formation (CWT) is used to get SWC measures as per [82, 83]. The SWC value at any date and for all considered frequencies, corresponding to the observations horizons varying between 1 and 64 days, always stays between zero and unity, being these boundaries representative of null and perfect positive cor relation bet ween the two analyzed arrays of data. In order to supplement our coherence setup and obtain a more profound knowledge regarding the leads-and-lags by the MCI and the yield-curve shaping parameters, the WCPD methodology [30, 59] is applied.

In what concerns the SWC methodology, it may be briefly described in the three consecutive phases. During the first step, the two analyzed time series,—*x* (*t*) and *y* (*t*),—are converted to their joint CWT [81], which may be written using their stand-alone CWTs, namely, $W_{n}^{x}(u,s)$ and $W_{n}^{y}(u,s)$ as:

$$W_{n}^{x}{}^{y}(u,s)=W_{n}^{x}{\left(u,s\right)}^{*}W_{n}^{y}(u,s),$$ (1)

where *u* denotes loc ation, *s* stands for scale, and the star * represents the complex conjugation. The joint CWT allows distinguishing the regions in the time–frequency space, characterized by co-movements between the two data arrays, even at occasions when their com mon high power is not observed. Putting it in different words, the joint CWT represents, at each scale, a local covariance of the datasets. E.g., a CWT figure near a unitary value signals that the two time–series are highly synchronized, while a CWT in a vicinity of zero indicates a lack of meaningful interrelation.

As the second step, the SWC expression is defined [82], which, being based on the joint and individual CWTs, continues to be representative of the dataset’s comovements:

$$R^{2}(u,s)=\frac{|S(s^{\left(-1\right)}W^{x}{}^{y}(u,s)){|}^{\left(2\right)}}{S(s^{\left(-1\right)}|W^{x}(u,s){|}^{\left(2\right)})S(s^{\left(-1\right)}|W^{y}(u,s){|}^{\left(2\right)})}.$$ (2)

Here S designates smoothing on time and frequency scale. One can interpret the SWC parameter as a correlation measure in the time–frequency space, with the respective range of values confined bet ween zero and unity.

However, in contrast with a well-known unidimensional Pear son coefficient, often used to describe a correlation between two arrays of data, which varies between -1 and 1, SWC values, by construction, belong to the [0, 1] interval. Therefore, this metrics cannot help to determine whether the analyzed comovements are of the same or of opposite signs, not being able to provide differentiation bet ween the two type of correlation, namely, positive and negative.

The third step in the development of the wavelet-based approach targets to gain additional knowledge in respect of correlation analysis and leads-and-lags patterns subjacent to the two considered arrays of data. With this purpose we adhere to the WCPD analysis [82], permitting to distinguish between positive and negative comovements. We utilize the following WCPD expression:

$$\Phi_{xy}(u,s)=tan^{-1}(\frac{Im\{S(s^{\left(-1\right)}W^{x}{}^{y}(u,s))\}}{Re\{S(s^{\left(-1\right)}W^{x}{}^{y}(u,s))\}}),$$ (3)

with *Re* and *Im* representing the real and imaginary parts of the joint smoothed CWT, respectively. A set of two data arrays with a null phase-difference is an example of perfectly co-moving time-series.

We use a standard visual depiction of the results, based on heatmap panels for both measures, namely, the SWC and the WCPD. Phase relation ships between the two time-series under consideration are represented in the SWC heatmaps by arrowheads. An arrow pointing to the right/left signifies that the data arrays behave in in-phase/anti-phase mode, corresponding to a positive/negative correlation between these time series. An arrow pointing downward/upward means that *y* (*t*) / *x* (*t*) leads *x* (*t*) / *y* (*t*) by π/2. Taking into account the above-given precepts, it is possible to easily interpret the message brought about by an arrow whatever is a direction it indicates.

In the following section we discuss how to estimate the yield-curve shaping parameters.

## 4. Estimation of the yield-curve shaping parameters

We stick to the methodology, developed in [62], which allows estimating the three yield-curve shaping parameters, namely the level, slope, and the curvature coefficients, which at any given moment in time define the shape of the term structure of interest rate with great accuracy. Although there are several econometric frameworks capable of fitting a yield curve [63], methodology has been gaining a predominant attention from academy because of the three following inherent characteristics, discussed in detail in [84]. First, the discount factor dependence on term maturity exhibits a declining tendency with the rise in maturity, with the discount coefficient becoming practically a zero multiplier for a very long end of the term structure. In this manner [62], approach perfectly fits the time-value-of-money requirements of the mainstream macroeconomic handbooks. As a second feature, we highlight that it is a parsimonious approach, the fact which results in its enhanced fore casting capacity. And the third, final but yet important peculiarity of this methodology is that it can be employed for estimating all and any kind of term structures, possessing market data necessary for their empirical estimation.

The original [62] model has been further enhanced in [63], in order to research the dynamic nature of term structures of interest rates. In [63], as well as in [85], it is argued that the dynamics of the yield-curve shaping parameters follows a vector–autoregressive structure of order one. This assumption permits estimating the yield-curve shaping parameters in a state–space framework. The state–space version of [63] econometric setup is specified in the following way (please refer to [63] for complete details);

$$z_{t}(\tau )={\left(\begin{array}{lll} 1 & \left(\frac{1-e^{-\lambda \tau_{1}}}{\lambda \tau_{1}}\right) & \left(\frac{1-e^{-\lambda \tau_{1}}}{\lambda \tau_{1}}-e^{-\lambda \tau_{1}}\right)\\ 1 & \left(\frac{1-e^{-\lambda \tau_{2}}}{\lambda \tau_{2}}\right) & \left(\frac{1-e^{-\lambda \tau_{2}}}{\lambda \tau_{2}}-e^{-\lambda \tau_{2}}\right)\\ \vdots & \vdots & \vdots \\ 1 & \left(\frac{1-e^{-\lambda \tau_{N}}}{\lambda \tau_{N}}\right) & \left(\frac{1-e^{-\lambda \tau_{N}}}{\lambda \tau_{N}}-e^{-\lambda \tau_{N}}\right) \end{array}\right)}^{\prime }x_{t}+u_{t},u_{t}∼N(0,R)$$

$${\tilde{x}}_{t}=\Gamma {\tilde{x}}_{t-1}+\eta_{t},\eta_{t}∼(0,G),$$ (4)

where *z*_*t*_(*τ*) denotes an N×1 dimensional vector for yields, *u*_*t*_ represents another N×1 vector, populated by error terms. ***x***_***t***_ **=[*L***_***t***_**,*S***_***t***_**,*C***_***t***_**]** represents a 3 × 1 dimensional vector containing the yield-curve shaping parameters. In particular, *L*_*t*_ denotes the level, *C*_*t*_ denotes the curvature, and *S*_*t*_ denotes the slope. Regarding the subsequent transition equation, ${\tilde{x}}_{t}=x_{t}-{\tilde{x}}_{t-1}$ stands as a representation for the matrix of the demeaned time–varying latent yield-curve shaping factors, Γ represents the dynamic interrelation of the latent factors shaping the term structure, *η*_*t*_ is a 3 × 1 dimensional array containing the respective errors. The convention is that *η*_*t*_ and *u*_*t*_ are independent. ***G*** represents an N×N diagonal matrix. In its turn, ***R*** stands for a 3 × 3 variance–covariance matrix.

The sample statistics of the level, slope, and the curva ture factors describing the term structures of the BRICS sovereign yields along with the descriptive analysis of the media coverage data are presented in the next section.

## 5. Data and descriptive statistics

We analyze the first ten months of the year 2020. We extract the historical data on the MCI behavior from the data analytics platform Raven pack. The Raven pack MCI gauges the Covid-19 cover age by social media as a percentage of news providers, addressing the Covid-19 themes and concerns, in relation to the total number of media content providers. In this way, the MCI is represent ative of the Covid-19 advancements and impacts on the general moral and state of mind in the society, as it captures the dynamic of the coronavirus awareness among investing agents. The range of MCI values is but the interval [0, 100]. Note that one hundred figure indicates the maximum degree of Covid-19 media coverage.

In addition to the MCI, we include in our data the time series of the three latent yield-curve shaping factors,—level, slope, and curva ture,—for each of five EM economies, members of BRICS. These yield-curve shaping factors are estimated for the five above-specified term structures by means of the widely used dynamic model, originally proposed in [62] and modified by [63]. For each country, we use the time series of the zero-coup on yields, at fifteen different points of the term structure. We consider the following terms: 0.25, 05, 1, 2, 3, 4, 5, 6, 7, 8, 9, 10, 15, 20, and 30-year points. The time series of sovereign interest rates are extracted via Bloomberg terminal.

Table 1 below represents the summary statistics for the daily changes for the level, slope and curvature time series derived from the sovereign yield-curves for BRICS from the beginning of January to the end of October 2020. We notice sizable differences across the components of yield curve among the BRICS countries. However, for all the three components South Africa exhibit the highest mean values, followed by Brazil. China exhibits the lowest means for all three parameters. Similarly, the standard deviation of the yield-curve shaping parameters is the highest for South Africa and Brazil and the lowest for China.

**Table 1:** Sample statistics: BRICS yield-curve shaping factors and the MCI.

	L_BRAZIL	L_CHINA	L_INDIA	L_RUSSIA	L_S_AFRICA	S_BRAZIL	S_INDIA	S_CHINA	S_RUSSIA	S_S_AFRICA	C_BRAZIL	C_CHINA	C_INDIA	C_RUSSIA	C_S_AFRICA	MCI
Mean	-6.55	-0.72	-3.23	-2.78	-13.31	-5.98	-3.31	-1.80	-1.84	-11.95	-6.55	-0.72	-3.23	-2.78	-13.31	64.55
Median	-6.57	-0.53	-3.46	-3.02	-13.96	-6.39	-3.67	-1.60	-1.70	-13.50	-6.57	-0.53	-3.46	-3.02	-13.96	73.74
Maximum	-3.84	0.66	-1.22	1.60	-7.53	-3.59	-2.02	-0.95	-0.22	-5.59	-3.84	0.66	-1.22	1.60	-7.53	82.95
Minimum	-10.04	-2.69	-4.93	-5.32	-18.16	-7.92	-4.14	-3.38	-3.20	-15.12	-10.04	-2.69	-4.93	-5.32	-18.16	0.09
Std. Dev.	1.40	0.73	0.86	1.17	3.30	1.23	0.68	0.63	0.90	3.25	1.40	0.73	0.86	1.17	3.30	21.52
Skewness	-0.24	-0.78	0.76	1.02	0.20	0.53	0.80	-1.11	-0.07	1.00	-0.24	-0.78	0.76	1.02	0.20	-1.81
Kurtosis	2.46	3.06	2.72	4.26	1.59	1.91	1.96	3.07	1.53	2.30	2.46	3.06	2.72	4.26	1.59	5.17
Jarque-Bera	4.66	21.55	21.25	50.66	18.99	20.48	32.13	43.83	19.16	39.95	4.66	21.55	21.25	50.66	18.99	157.06
Probability	0.10	0.00	0.00	0.00	0.00	0.00	0.00	0.00	0.00	0.00	0.10	0.00	0.00	0.00	0.00	0.00
Observations	212	212	212	212	212	212	212	212	212	212	212	212	212	212	212	212

*Comments*. This table documents changes observed on the daily basis in the BRICS yield-curve shaping factors and the MCI for Jan-Oct 2020. L_COUNTRY, S_COUNTRY, and C_COUNTRY stand, respectively, for the level, slope, and the curvature of the term structure of the yield rates of COUNTRY´s governmental debt.

## 6. Results

### 6.1. Media Cover age Index (MCI) and sovereign yield factors for Brazil

As the first step, we analyze the outcomes, obtained by means of the employed wavelet approach for the SWC between the MCI and latent yield-shaping level parameter for the Brazil term structure (Level_Brazil). The legends, placed to the right of the respective heatmaps help interpreting the meaning of the graphic color panels. The horizontal axis is a time scale and the vertical axis gauges frequency or investment/observation horizon. We interpret the presented graphs analyzing the color observable at any point in time and for any frequency within the considered range. Warmer colors,—red, yellow,—signify higher coherence, i.e., stronger interrelation of the time series. Cooler colors,—blue, green,—signal lower coherence.

**Fig 1:**
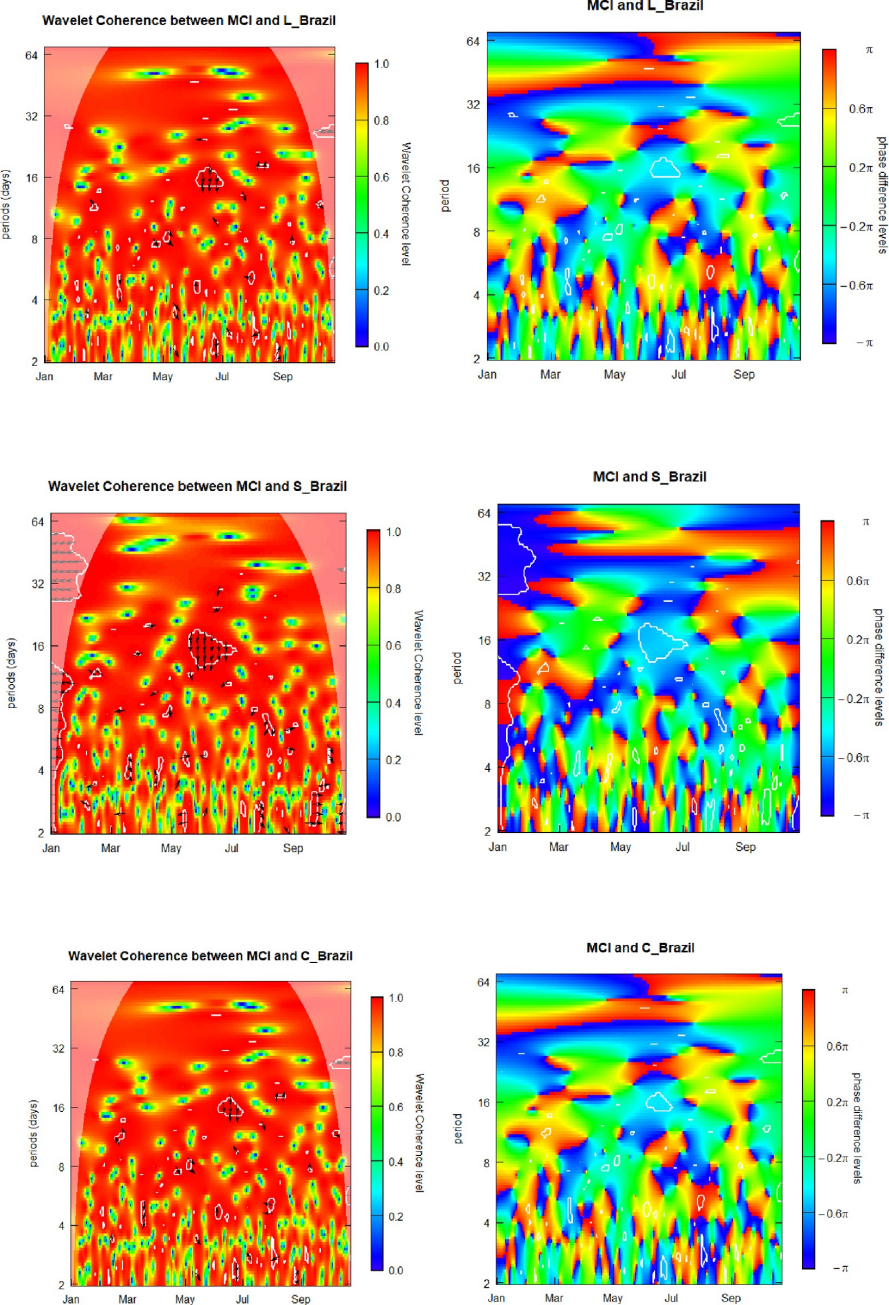
Media Coverage Index (MCI) and sovereign yield components of Brazil. L_Brazil, S_Brazil, and C_Brazil denote the level, curvature and slope component for Brazil.

Fig 1 below presents graphic visualizations of the SWC and WCPD metrics subjacent to the MCI and yield components of Brazil. Focusing on the MCI-Level_Brazil interrelation, we find that coherence levels vary along the time and across the frequencies alternating between low and high for the whole range of analyzed dates and investment/observation horizons. We highlight that, in the left heatmap, for the frequencies in the 3-week-plus band, from August onwards, the panel appears as preponderantly reddish, signifying an elevated coherence for the specified time-frequency region. However, we observe the two more pronounced blue spots with the green-to-yellow aureoles around late April and early July for approximately 1,5-month investment horizon. The first of them we attribute to the echo of the apogee of the Covid-19 crisis represented by credit crunch and liquidity squeeze, observed in late March on a global scale, when Brazil 10Y sovereign bond yield climbed to the levels above 9,5%. However, we posit that the nature of the second region of low coherence is idiosyncratic and ascribe it to the aftershocks of the pandemic pick, related to the specific country´s difficulties to revert the expansion of the virulent disease, which made Brazil 10Y sovereign bond yield, after initial decrease in April below 7%, newly climb above 8,5% and 8% in late April and early March, respectively.

We also observe that, along the whole analyzed period, the weakest level of coherence corresponds to the three-day’s frequency period. This finding seems to be quite relevant in that what concerns the Level_Brazil behavior, especially taking into consideration that the weak coherence areas observed in the range of low frequencies is not stable along the time scale, but represents a kind of alternating dynamics. It seems to be justifiable to continue a more advanced investigation this phenomenon.

Even though the preponderantly reddish-colored regions signal that Level_Brazil volatility is strongly correlated with the pandemic-triggered panicky sentiment that diminishes a possibility of diversifying risk exposures by means of using investment strategies involving this yield-curve factor, several greenish areas, observed in the heat map, signify that Level_Brazil is capable of providing certain diversifying potential, resilient to the severe impacts of Covid-19 contingency measures, undertaken by Brazilian government in the end of the first quarter of 2020.

The next step of our study is focused on the analysis of causality relations and phase—difference patterns of the Level_Brazil and MCI time series. Now we swiftly recall the meaning of the arrowheads in the SWC heatmaps as previously explained in more detail in Section 3 describing the wavelet econometric framework. Arrowheads in the SWC panel represent phase-difference relation of t he movements in the MCI and Level_Brazil time series. E.g., arrowheads ← or → signal that both, MCI and the level factor move, respectively, in anti-phase or in-phase. In addition, arrows ↑ and ↓ signify that the MCI, respectively, leads or is lagging the level factor of Brazil yield curve by π/2.

The heatmap at the left side of Fig 1, for the band comprising the 2–to–3-week frequencies, exhibits, in June, a cloud of the arrows ↓, indicating that the Level_Brazil time series leads the MCI. This observed lead by the Level_Brazil factor corresponds to the two-months decrease in sovereign yields, occurred form mid-May to mid-July and characterized by relatively low volatility, while the MCI revealed a rather side-trend behavior, being the overall Brazil improvement ahead of the relatively less expressive general recovery on a global scale.

In order to obtain additional knowledge about the interrelation of the analyzed pair of the variables, the WCPD panel helps us to determine the regions either of leads or lags, if any, relative to the MCI and Level_Brazil time series. Our four relevant findings are discussed herein. First, for the band comprising 4-to-5-weeks´ frequencies, a blue anti-phase belt covering the first eight months of 2020 is clearly observable in the panel, overtly evidencing a lead by the level factor of Brazil yield curve over the MCI along both the escalation of the Covid-19 crisis and initial recovery from it; after that the relation changes for synchrony since September. Second, we observe the region of anti-phase in March for 4-to-9-day frequency band, signifying that the level factor was more sensitive to the advancement and the retreat of the pandemic around it apogee in late March, characterized by the lockdown policies both worldwide and in Brazil. Third, interestingly enough, for the 7-days investment horizon we observe the 2-months long blue antiphase region, since mid-June to mid-August, coinciding with the all-Covid-19 lows of Brazil 10Y yield below 6,5% in late July and early August. Fourth, within the 1-to-4-days frequency range we notice interchanging style of the colored regions indicating un synchronized behavior of the time series. However, for lower frequencies in the 2-weeks-plus range, such a synchronies seem to be rather self-cancelled, revealing a predominant relationship of leading, lagging, or synchronized behavior. The latter outcome is especially useful for investing agents, who assess relative attractive ness of invest ments along the Brazil sovereign yield curve, as its latent level factor seemingly possesses some appealing hedge characteristics, whose potential for downside risk hedge strategies has been shown to remain through the global crisis based on the study of Covid-19 pandemic fueled meltdown, especially for around-1-month long investment horizons.

For the MCI-Slope_Brazil pair, similarly to the previously discussed case of MCI-Level_Brazil relation, we show that coherence levels vary along the time and across the frequencies alternating between low and high for the whole range of analyzed dates and investment/observation horizons. For the vertical range comprising the 2–week–plus frequencies we observe preponderantly reddish color, signifying a high coherence between the two time series over the entire analyzed period. We observe for the 4-weeks-plus investment horizons several well-pronounced blue spots with the green-to-yellow aureoles. This indicates, that in comparison with the level yield factor, the Slope_Brazil appears to be more sensitive to the macroeconomic conditions influenced by the pandemic media coverage.

Similarly to the Level_Brazil case, the weakest coherence we observe at the 3-day’s investment/observation horizon. However, the time-frequency locations of antiphase blue spots for Slope_Brazil differ from locations of such spots for Level_Brazil, indicating diversifications opportunities potentially existing between these two latent yield-curve factors.

The next step of our study is focused on the analysis of causality relations and phase—difference patterns of the Slope_Brazil and MCI time series. For the band comprising the 2–to–3–week frequencies, in late May and June, similarly to the Level_Brazil case, we observe a region of the pointing downward arrowheads, ↓, implying that the Slope_Brazil time series leads the MCI. We associate this lead by the Slope_Brazil factor to the two-months decrease in sovereign 10Y yield and simultaneous flattering of the sovereign yield curve.

In addition, differently form the Level_Brazil case, in January, we observe the clouds of arrowheads pointing to the left, ←, signaling an anti-phase interrelation for the 2-days-to-2-week frequency range and the 4-weeks-plus band, indicating the negative correlation bet ween the MCI and the Slope_Brazil. We ascribe this period to the early phase of the pandemic, when Covid-19 begins to spread on a global scale, when the increase in the pandemic media coverage coincides with the decrease of the yields and flattering of the yield curve of Brazil.

In order to obtain additional knowledge about the interrelation of the analyzed pair of the variables, the WCPD panel helps us to determine the regions either of leads or lags, if any, relative to the MCI and Slope_Brazil time series. Although the overall pattern of this panel somewhat resembles the pattern of the corresponding WCPD Level_Brazil heatmap, we observe two important features, different from the Level_Brazil phase-difference heatmap. First, the overall green area, representing a synchronized behavior of the two analyzed time series in the Slope_Brazil case is considerably reduced in comparison with the Level_Brazil heatmap, indicating that the slope factor potentially represents more diversification attributes than the level factor of the Brazil yield curve. Second, it is worth mentioning that the borders between the deep blue regions, on one hand, and the green and intense red zones, on the other hand, are quite neat, serving as an indication of lead-lags regime switching. These observed breakdowns of the MCI-Slope_Brazil relations represents a valuable attribute for creating elaborated dynamic hedge strategies based on investments in the government debt of Brazil at different maturities.

The latter result is potentially relevant for investment professionals, who assess attractiveness of debt exposures along the Brazil sovereign yield curve, as its latent slope factor seemingly possesses appealing hedge features, whose capacities to serve as a base for downside risk hedge strategies, as we have evidenced based on the Covid-19 meltdown, remains present through the global crises, especially for around-1-month long investment horizons.

Lastly, the MCI-Curvature_Brazil patterns are very similar to those of the level and slope panels in Fig 1. Hence, the corresponding results concerning the interrelation between the MCI and volatility of the component Curvature_Brazil are to be the same as in the MCI-Level_Brazil case. Therefore, we evidence that there is no potential for level-curvature cross-factors hedge strategies. We also argue that it is not reasonable to design hedge strategies based on curvature yield factor, as the same result could be obtained by using the level yield factor with much less mathematical and trade efforts. Then, we do not believe that the curvature component of the Brazil term structure represents an eligible candidate for hedge strategies design due to similarity of its behavior, along the Covid-19 crisis, to the behavior of the level yield factor, while eventual employment of the curvature would bring about an unjustified mathematical and practical complexity.

### 6.2. Media Coverage Index (MCI) and sovereign yield factors for Russia

**Fig 2:**
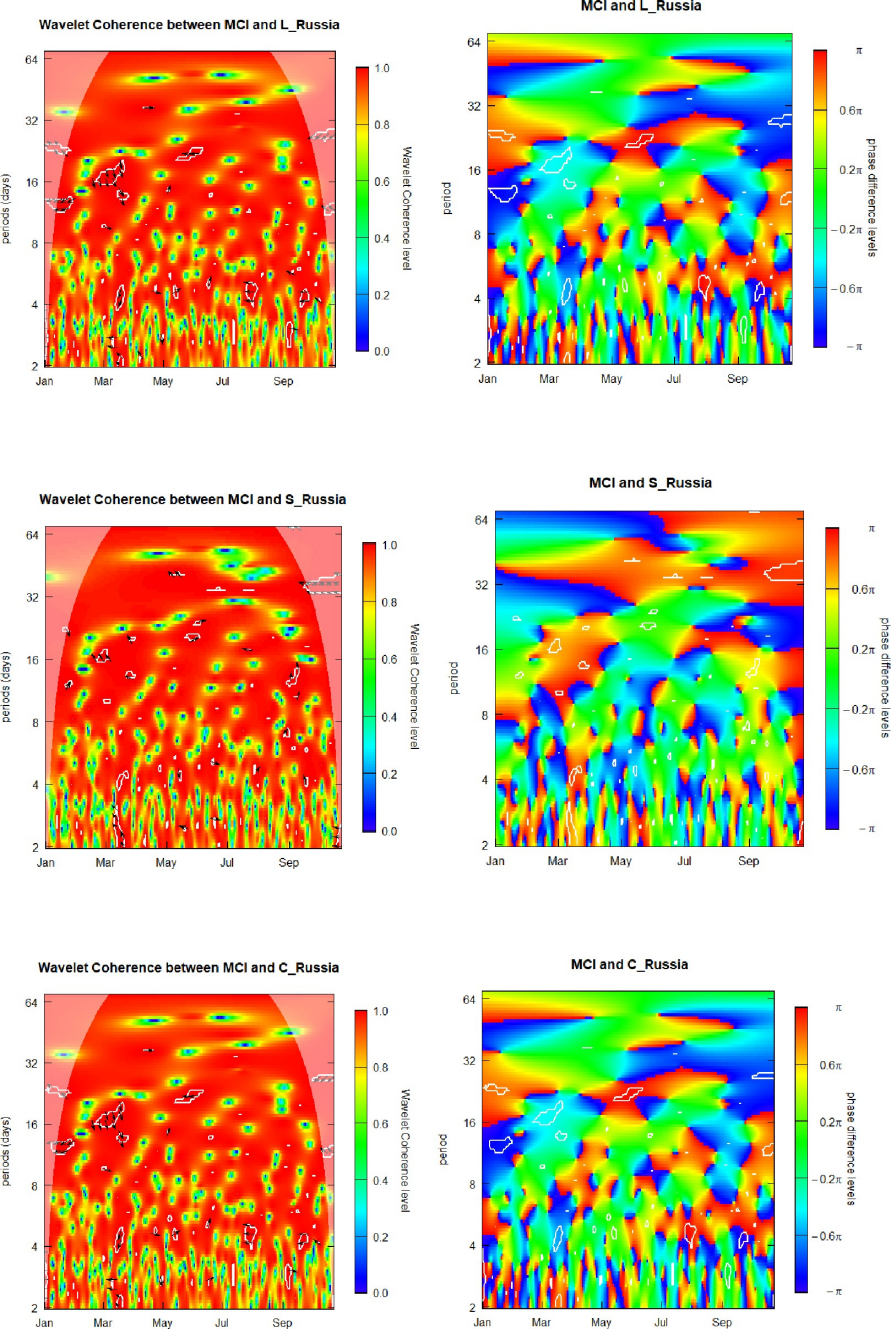
Media Coverage Index (MCI) and sovereign yield components of Russia. L_Russia, S_Russia, and C_Russia denote the level, curvature and slope component for Russia.

Now our study advances further on and we discuss the findings regarding the relation between the MCI and the three latent yield factors of the Russian sovereign curve. Fig 2 presents graphic visualizations of the SWC plus WCPD metrics subjacent to the MCI and yield components of Russia.

For the MCI-Level_Russia pair, as in the previously discussed case of the MCI-Level_Brazil pair, we show that coherence levels vary along the time and across the frequencies alternating between low and high for the whole range of analyzed dates and investment/observation horizons. The overall MCI-Level_Russia patterns exhibited in the left panel of Fig 2 above are similar to those of the left heatmap of Fig 1. Hence, the corresponding conclusions on the MCI-Level_Russia coherence are similar to those in the case of the MCI-Level_Brazil pair.

However, differently from the MCI-Level_Brazil case, in the left heatmap of Fig 4, for the 2-to-3-weeks frequency band, in late February and early March, we observe the cloud of the ↘ arrows, indicating that the two data arrays behave in-phase and that the Level_Russia time series is leading the MCI. The observed herein lead by the Level_Russia factor corresponds to the period of major escalation of the pandemic-fueled crisis just prior to its apogee in late March, when sovereign yields of Russia have been more susceptive to changing economic conditions than the coverage of the pandemic expansion by the media.

In order to obtain additional knowledge about the interrelation of the analyzed pair of the variables, the WCPD panel helps us to determine the regions either of leads or lags, if any, relative to the MCI and Level_Russia time series. Our three relevant findings are discussed herein. First, the boundaries separating the deep blue areas, on one hand, and the green and intense red zones, on the other hand, are quite neat, serving as an indication of lead-lags regime switching. These observed breakdowns of the MCI-Level_Russia relations represent a valuable attribute for creating elaborated dynamic hedge strategies based on investments in the government debt of Russia at different maturities. Second, the regime switching is especially well evidenced along the frequency scale by intermittent blue and red regions in both, left and right sides of the heatmap. These time intervals correspond, respectively, to the initial expansion of the pandemic in January and to the escalation of the second wave in October. Third, the time-frequency locations of green zones indicating a synchronized behavior of the media-level pair, as well as locations of out-of-phase blue and red zones, differ considerably for the MCI-Level_Russia (left panel, Fig 2) and MCI Level_Brazil (left panel, Fig 1) cases. This certifies an attractive cross-country diversification potential for investment strategies based on the level factor of the yield curves of Russia and Brazil.

For the MCI-Slope_Russia pair, similarly to the previously discussed cases, we show that coherence levels vary along the time and across the frequencies alternating between low and high for the whole range of analyzed dates and investment/observation horizons. For the vertical range comprising the 2–week–plus frequencies we observe preponderantly reddish color, signifying a high coherence between the two time series throughout the whole analyzed interval. We observe the weakest coherence at the 3-days investment horizon. However, pattern of the SWC heatmap for the MCI-Slope_Russia pair is considerably different, even visually, from the pattern of the SWC heatmap for the MCI-Level_Russia pair, indicating that there exists an attractive diversification potential to be considered in designing cross-factor hedge strategies involving investment in Russian governmental debt at different maturities.

In order to obtain additional knowledge about the interrelation of the analyzed pair of the variables, the WCPD panel helps us to determine the regions either of leads or lags, if any, relative to the MCI and Slope_Russia time series. We observe two important features. First, the green in-phase areas of the MCI-Slope_Russia phase-difference heatmap (Fig 2, right panel) almost perfectly correspond to the out-of-phase blue and red areas of the MCI-Level_Russia phase-difference heatmap (Fig 1, right panel) except for October, and, vice versa, the green in-phase areas of the MCI-Level_Russia phase-difference heatmap (Fig 2, right panel) almost perfectly correspond to the out-of-phase blue and red areas of the MCI-Slope_Russia phase-difference heatmap (Fig 2, right panel). This serves as a confirmation of a considerable diversification potential, capable of benefiting cross-factor hedge strategies involving investment in Russian governmental debt at different maturities. Second, the borders between the deep blue regions corresponding to the Slope_Russia leading the MCI and the intense red zones, where Slope_Russia is lagging behind the MCI, are quite neat, especially in October, closed to the right-hand border of the panel, that serves as an indication of lead-lags regime switching. These observed breakdowns of the MCI-Slope_Russia lead-lag relations represent a valuable attribute for creating elaborated dynamic hedge strategies based on investments in the government debt of Russia at different maturities.

The latter result is potentially relevant for investment professionals, who assess attractive ness of debt exposures along the Russia sovereign yield curve, as its latent slope factor seemingly possesses appealing hedge features, whose hedge-strategy potential for the whole range of investment horizons, remains present through the global crises, as we have evidenced based on the Covid-19 meltdown.

Lastly, we discuss the MCI and Curvature_Russia pair. The overall MCI-Curvature_Russia patterns are very similar to those of the heatmaps of level and slope. Hence, the corresponding conclusions concerning the interrelation between the MCI and volatility of the Curvature_Russia component are to be the same as in the MCI-Level_Russia case. Therefore, we evidence that there is no potential for level-curvature cross-factors hedge strategies. We also do not believe that it is reasonable to design hedge strategies based on curvature yield factor, as the same result could be obtained by using the level yield factor with much less mathematical and portfolio management efforts. Hence, we do not believe that the curvature component of the yield-curve of Russia is an eligible candidate for hedge strategies design due to similarity of its behavior, along the Covid-19 crisis, to the behavior of the level yield factor, while eventual employment of the curvature would bring about an unjustified mathematical and practical complexity.

### 6.3. Media Cover age Index (MCI) and sovereign yield factors for India

At this point we begin discussing report our outputs regarding the relation between the MCI and the three latent yield factors of the India sovereign curve.

**Fig 3:**
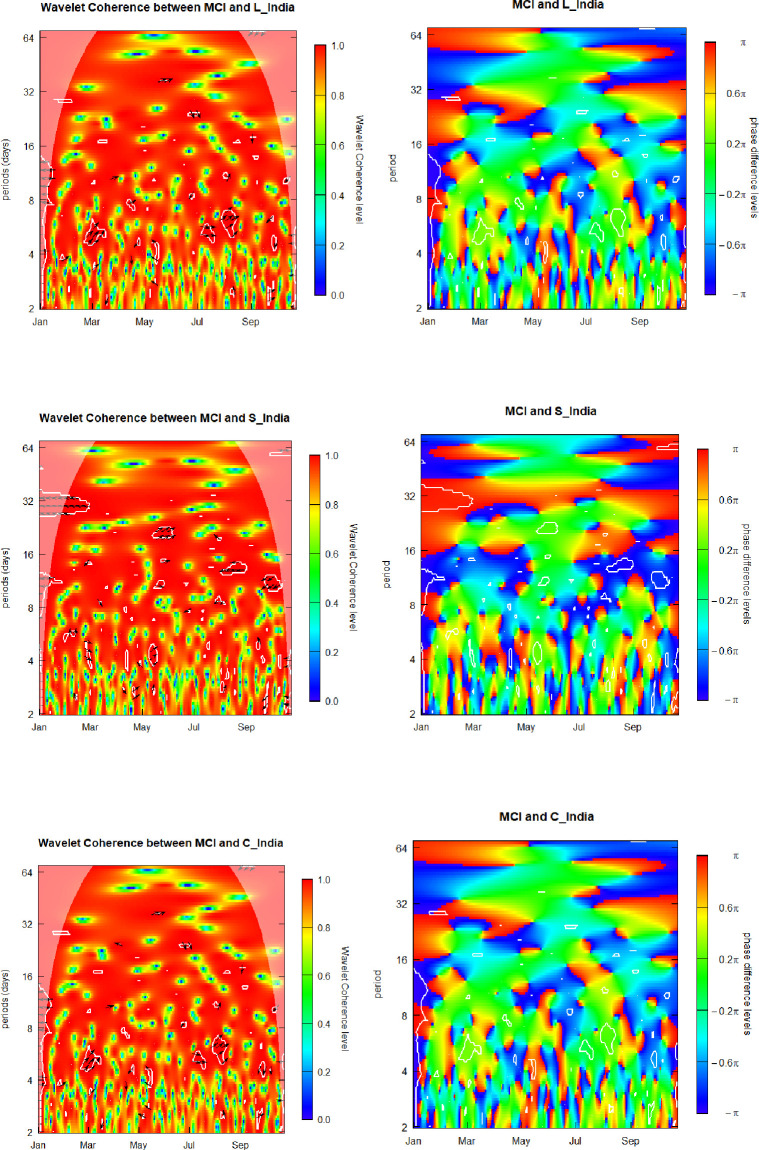
Media Coverage Index (MCI) and sovereign yield components of India. L_India, S_India, and C_India denote the level, curvature and slope component for India.

Fig 3 presents graphic visualizations of the SWC plus WCPD metrics subjacent to the MCI and yield-curve components for India. For the MCI-Level_India pair, as in the previously discussed cases of the MCI-Level_Brazil and MCI-Level_Russia pairs, we show that coherence levels vary along the time and across the frequencies alternating between low and high for the whole range of analyzed dates and investment/observation horizons. The overall MCI-Level_India patterns exhibited in the left panel of Fig 3 are similar to those of the left heatmaps of Figs 1 and 2. Hence, the corresponding conclusions concerning the MCI-Level_India coherence are similar to those in the case of the MCI-Level_Brazil and MCI-Level_Russia pairs.

However, differently to the previous cases, on the left side of the SWC heatmap of Fig 3, for the 2-to-14-days frequency band, we notice a cloud of the left-oriented arrowheads, ←, implying a clear anti-phase behavior and indicating negative correlation between the MCI and Level_India factor. We attribute this phenomenon to the early phase of the coronavirus outbreak, characterized by a rapid propagation of this virulent disease across diverse regions and countries, when the dynamics of the India sovereign yield curve was not yet synchronized with changes in the pandemic media coverage.

In order t o obtain additional knowledge about the interrelation of the analyzed pair of the variables, the WCPD panel of Fig 3 helps us to determine the regions either of leads or lags, if any, relative to the MCI and Level_India time series. We observe three important features. First, the frontiers separating the deep blue regions from the intense red zones appear as quite neat, serving as an indication of lead-lags regime switching. These observed breakdowns of the MCI-Level_India relations represent an attractive attribute for creating elaborated dynamic hedge strategies based on investments in the government debt of India at long maturities, as the level factor represents the behavior of the interest rates at the long end of the yield term-structure. Second, the regime switching is especially well evidenced along the frequency scale by intermittent blue and red regions on both, left and right sides of the heatmap. These time intervals correspond, respectively, to the initial expansion of the pandemic in January and to the escalation of the second wave in October. Third, in the center of the heat map we observe three well-pronounced deep blue regions: in April-May for the 2-to-3-weeks frequency band, in May-June for the 1-to-2-weeks frequencies, and in August, for the 2-to-3-weeks investment horizons. The first and the third blue regions correspond to the local maxima of the Indian 10Y yield, while the second blue region corresponds to the all-Covid-19 local minimum reached on July 10, when clearly idiosyncratic moves of India yield curve were ahead of changes in the pandemic media coverage.

Our empirical observations are potentially insightful for investment communities, which assess attractive ness of investing investments in India governmental debt at long maturities, as its latent level factor seemingly possesses appealing to investors hedge properties, whose capacity to serve as a base for downside risk hedge strategies, as we have evidenced based on the Covid-19 meltdown, remains present through the global crises.

For the MCI-Slope_India pair, similarly to the previously discussed cases, we show that coherence levels vary along the time and across the frequencies alternating between low and high for the whole range of analyzed dates and investment/observation horizons. For the vertical range comprising the 2–week–plus frequencies we observe preponderantly reddish tonality, signifying a high coherence between the two time series throughout the whole analyzed interval. The weakest coherence level corresponds to the 3-days investment horizon. However, the pattern of the SWC heatmap for the MCI-Slope_India pair differs from the pattern of the SWC heatmaps for the MCI-Slope_Brazil and MCI-Slope_Russia pairs. It also differs from the pattern of the MCI-Level_India pair. These two features indicate that there exists an attractive diversification potential to be considered in designing cross-country as well as cross-factor hedge strategies involving investment in Indian governmental debt at different maturities.

In order to obtain additional knowledge about the interrelation of the analyzed pair of the variables, the WCPD panel helps us to determine the regions either of leads or lags, if any, relative to the MCI and Slope_India time series. We observe four important features. First, the overall green area, representing synchro nized behavior of the two analyzed time series in the Slope_India case, is considerably reduced in comparison with the Level_India heatmap, indicating that the slope factor potentially represents more diversification attributes while compared to the level factor of the Indian yield curve. Second, for investment horizons about one month, we observe an intensive red belt along the whole studied period, indicating the continuous lead of the MCI over the Slope_India component of the term structure. Third, the borders separating the deep blue regions corresponding to the Slope_India leading the MCI and the intense red zones, where Slope_India is lagging behind the MCI, are quite neat, serving as an indication of lead-lags regime switching. Fourth, the regime switching is especially well evidenced along the frequency scale by intermittent blue and red regions on both, left and right sides of the heatmap. These time intervals correspond, respectively, to the initial expansion of the pandemic in January and to the escalation of the second wave in October.

These outcomes are potentially relevant for investment professionals, who assess attractiveness of debt exposures along the India sovereign yield curve, as its latent slope factor seemingly possesses appealing hedge features, whose hedge-strategy potential for the whole range of investment horizons, remains present through the global crises, as we have evidenced based on the Covid-19 meltdown.

The overall MCI-Curvature_India patterns exhibited in the last two panels of Fig 3 are very similar to those of the two heatmaps at the top of this figure. Hence, the corresponding conclusions concerning interrelations between the MCI and volatility of the Curvature_India, are to be the same as in the MCI-Level_India case. Therefore, we evidence that there is no potential for level-curvature cross-factors hedge strategies. We also do not believe that it is reasonable to design hedge strategies based on curvature yield factor, as the same result could be obtained by using the level yield factor with much less mathematical and portfolio management efforts. Thus, we argue that the curvature of the Indian term structure does not represent an eligible candidate for hedge strategies design due to similarity of its behavior, along the Covid-19 crisis, to the behavior of the level yield factor, while eventual employment of the curvature would bring about an unjustified mathematical and practical complexity.

### 6.4. Media Coverage Index (MCI) and sovereign yield factors for China

We continue presenting our outcomes by discussing findings regarding the relation between the MCI and the three yield-curve shaping parameters for the yield term structure of China governmental debt.

**Fig 4:**
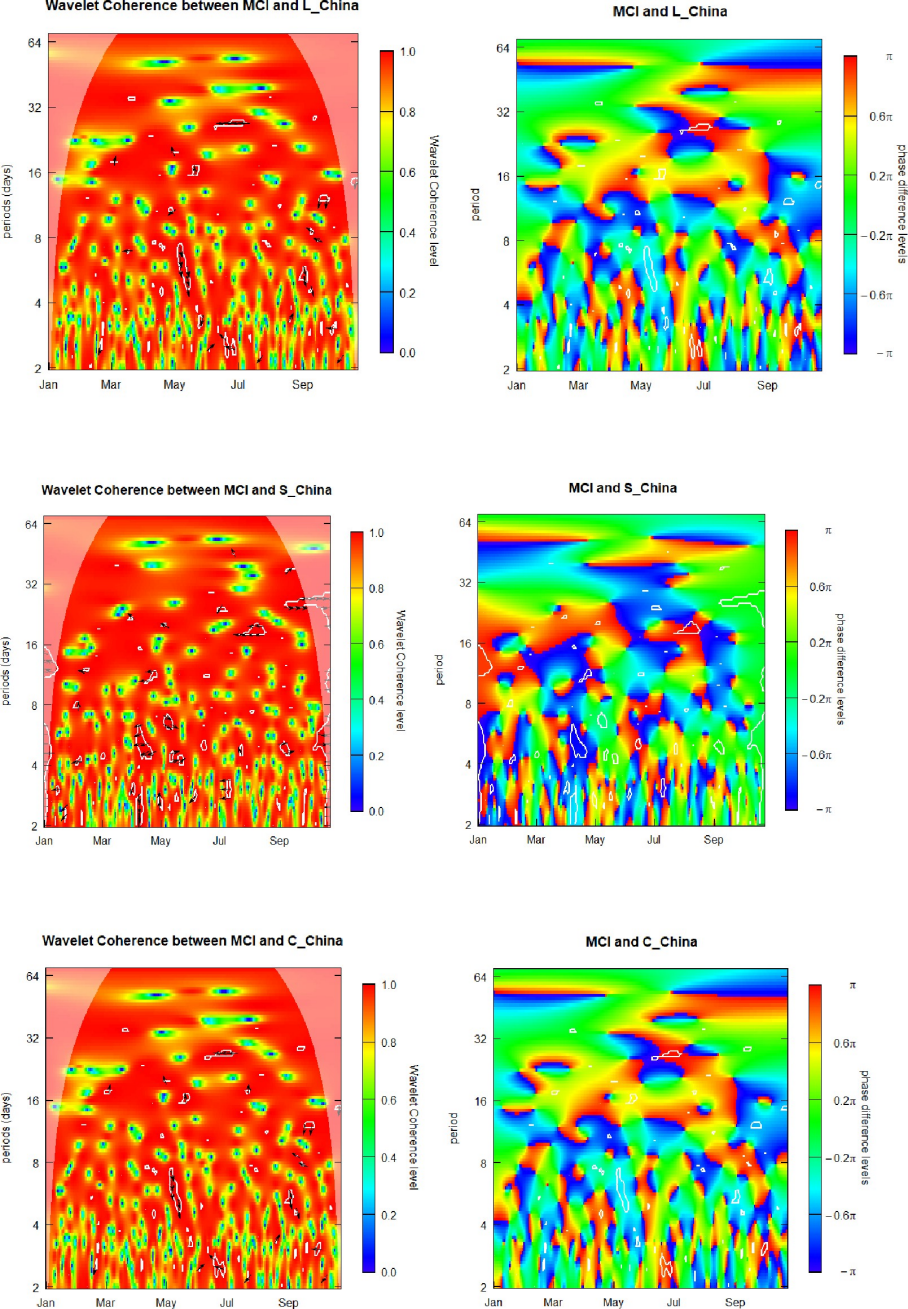
Media Coverage Index (MCI) and sovereign yield components of China. L_China, S_China, and C_China denote the level, curvature and slope component for China.

Fig 4 presents graphic visualizations of the SWC plus WCPD metrics subjacent to the MCI and yield-curve components for China. For the MCI-Level_China pair, as in the previously discussed level-factor and media coverage interrelations, we show that coherence levels vary along the time and across the frequencies alternating between low and high for the whole range of analyzed dates and investment/observation horizons. The overall MCI-Level_China patterns exhibited in the left panel of Fig 4 are similar to those of the left heatmaps of Figs 1–3. Hence, the respective findings regarding the MCI-Level_China coherence are similar to those in the case of the MCI-Level_Brazil, MCI-Level_Russia, and the MCI-Level_India pairs.

In order to obtain additional knowledge about the interrelation of the analyzed pair of the variables, the WCPD panel of Fig 4 helps us to find the regions either of leads or lags, if any, relative to the MCI and Level_China time series. We observe three important features. First, the boundaries separating the deep blue regions and intense red zones are quite neat, serving as a clear indication of lead-lags regime switching across diverse locations in the time-frequency space. These observed breakdowns of the MCI-Level_China relations represent an attractive attribute for creating elaborated dynamic hedge strategies based on investments in the government debt of China at long maturities. Second, the regime switching is especially well pronounced in the central region of the panel, which corresponds to high volatility and the short-term trends alternations of Chinese yields, starting to upsurge from the pandemic-caused March 2020, meltdown onwards and ending in September 2020. Third, the center of the heatmap exhibits predominantly warm colors, from yellow to red, meaning that the MCI leaded the changes in Level_China for the 2-to-3 weeks investment horizons since late March until September. It makes sense as Chinese economy is heavily depended on the global economy, and, hence, during the drastic moments follows the global news on pandemics.

The latter feature is potentially insightful for investment professional, especially those who analyze relative attractive ness of investing in China governmental debt at long maturities, as its latent level factor seemingly is capable of providing appealing hedge properties, suitable for designing downside risk hedge strategies, as we have evidenced based on the Covid-19 meltdown, remains present through the global crises.

For the MCI-Slope_China pair, similarly to the previously discussed cases, we show that coherence levels vary along the time and across the frequencies alternating between low and high for the whole range of analyzed dates and investment/observation horizons. For the vertical range comprising the 2–week–plus frequencies we observe preponderantly reddish tonality, signifying a high coherence between the two time series throughout the whole analyzed interval. We observe the weakest level of coherence at the 3-days investment horizon. However, the pattern of the SWC heatmap for the MCI-Slope_China pair somewhat differs from the previously discussed slope-factor heatmaps, indicating that there exists an attractive diversification potential to be considered in designing cross-country hedge strategies involving investment in China governmental debt at different maturities.

In order to obtain additional knowledge about the interrelation of the analyzed pair of the variables, the WCPD panel helps us to determine the regions either of leads or lags, if any, relative to the MCI and Slope_China time series. We observe three important features. First, the distribution of the warm, neutral green, and cool colored areas are different form the phase difference heatmap for the MCI and Level_China time series. This result certifies a potential possibility to design cross-factor hedging strategies based on investments in sovereign debt of China. Second, the frontiers separating the deep blue areas corresponding to the Slope_China leading the MCI and the intense red zones, where Slope_China is lagging behind the MCI, are quite neat, serving as an indication of lead-lags regime switching. Third, we also observe, in October, a vertical green belt, indicating in-phase behavior of the two time series. We posit that this feature indicates that the Covid-19 crisis has been mostly overcome in China, and that the rapid recovery has turned to rather normal economic activity, which is obviously sensitive to the health of the world economy as represented by the Covid-19 media coverage.

These findings seem to be insightful for investing agents, interested to correctly assess relative attractiveness of investing along the China sovereign yield curve, as its latent slope factor clearly possesses appealing hedge properties, suitable for designing hedging strategies workable across the whole range of investment horizons, and which remain present through the global crises, as we have evidenced based on the Covid-19 meltdown.

The overall MCI-Curvature_China patterns, exhibited in the last two panels of Fig 4, are very similar to those of the heatmaps of the first two panels of this figure. Hence, the corresponding conclusions concerning interrelations between the MCI and volatility of the Curvature_China component are to be the same as in the MCI-Level_China case. Therefore, we evidence that there exists no potential for level-curvature cross-factors hedge strategies. We also do not believe that it is reasonable to design hedge strategies based on curvature yield factor, as the same result could be obtained by using the level yield factor with much less mathematical and portfolio management efforts. Thus, we do not believe that the curvature parameter of the China term structure represents an eligible candidate for hedge strategies design due to similarity of its behavior, along the Covid-19 crisis, to the behavior of the level yield factor, while eventual employment of the curvature would bring about an unjustified mathematical and practical complexity.

### 6.5. Media Coverage Index (MCI) and sovereign yield factors for South Africa

We finalize our discussion by presenting the outcomes regarding the relation between the MCI and the three yield-shaping parameters of the South African sovereign term structure.

**Fig 5:**
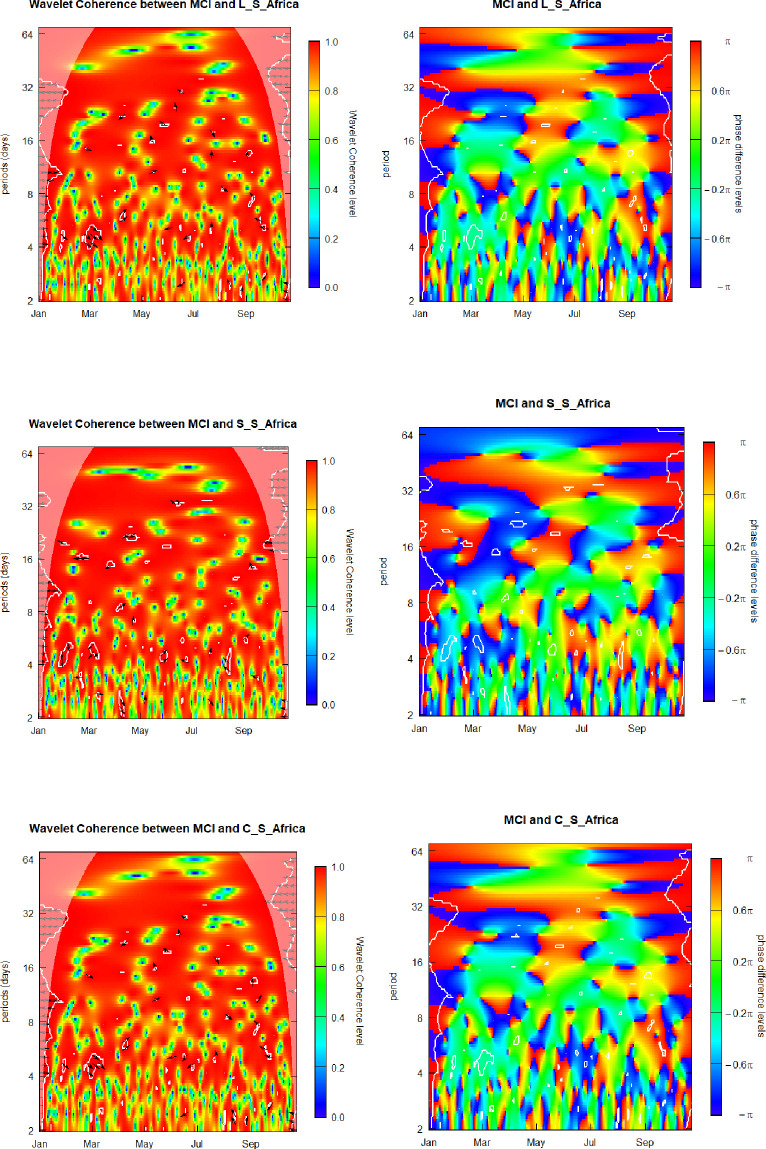
Media Coverage Index (MCI) and sovereign yield components of South Africa. L_S_Africa, S_S_Africa, and C_S_Africa denote the level, curvature and slope component for South Africa.

Fig 4 presents graphic visualizations of the SWC plus WCPD metrics subjacent to the MCI and yield-curve components for South Africa. For the MCI-Level_S_Africa pair, as in the previously discussed level-factor and media coverage interrelations, we show that coherence levels vary along the time and across the frequencies alternating between low and high for the whole range of analyzed dates and investment/observation horizons. The overall MCI-Level_S_Africa patterns exhibited in the left panel of Fig 5 are similar to those of the left heatmaps of Figs 1–4. Hence, the respective findings regarding the MCI-Level_S_Africa coherence are similar to those in the case of the MCI-Level_Brazil, MCI-Level_Russia, MCI-Level_China, and the MCI-Level_India pairs.

In addition, similarly to the MCI-Level_India case, in the left-hand side of the SWC heatmap of Fig 5, for the 2-to-35-days frequency band, we see a cloud of left-pointing arrowheads, ←, signaling an anti-phase behavior and indicating negative correlation between the MCI and the Level_S_Africa factor. It seems plausible to attribute this phenomenon to the early phase of the coronavirus outbreak, characterized by a rapid propagation of this virulent disease across diverse regions and countries, when the dynamics of the South African sovereign yield curve was not yet synchronized with changes in the pandemic media coverage.

In order to obtain additional knowledge about the interrelation of the analyzed pair of the variables, the WCPD panel of Fig 5 helps us to find regions either of leads or lags, if any, relative to the MCI and Level_S_Africa time series. We observe two important features. First, the boundaries separating the deep blue regions and warm reddish zones, are quite neat, serving as an indication of lead-lags regime switching across frequency scale especially closed to the left and right borders of the panel, corresponding respectively to the initial spread of the disease on the global scale and to the second wave, respectively. These observed breakdowns of the MCI-Level_S_Africa relations represent an attractive attribute for creating elaborated dynamic hedge strategies based on investments in the government debt of South Africa at long maturities. Second, the red belt for the 4-to-6-weeks frequency band spreads along the whole observation period, meaning that the MCI is leading alterations of the level component for the South Africa sovereign yield curve.

These features are capable of providing potential insights to investing professionals, who may be interested to correctly assess attractive ness of exposures to South African governmental debt at long maturities, as its latent level factor seemingly possesses attractive hedging attributes, although not for all frequency bands. Hence, the considered level factor reveals its potential for downside risk hedge strategies, as we have evidenced based on the Covid-19 meltdown, remains present through the global crises.

For the MCI-Slope_S_Africa pair, similarly to the previously discussed cases, we show that coherence levels vary along the time and across the frequencies alternating between low and high for the whole range of analyzed dates and investment/observation horizons. For the vertical range comprising the 2–week–plus frequencies we observe preponderantly reddish tonality, signifying a high coherence between the two time series throughout the whole analyzed interval. We observe the weakest coherence at the 3-days investment horizon. However, the pattern of the SWC heatmap for the MCI-Slope_S_Africa is somewhat different from the previously discussed slope-factor heatmaps, indicating that there is an attractive diversification potential to be considered in designing cross-country hedge strategies involving investment in South African governmental debt at different maturities.

In order to obtain additional knowledge about the interrelation of the analyzed pair of the variables, the WCPD panel helps us to determine the regions either of leads or lags, if any, relative to the MCI and Slope_China time series. We observe four important features. First, in the Slope_S_Africa case, the overall green area, representing synchro nized behavior of the two analyzed herein time series, is considerably reduced in comparison with the Level_S_Africa phase-difference heatmap, indicating that the slope factor potentially represents more diversification attributes than the level parameter of the South Africa term structure. Second, several edges separating the deep-blue regions corresponding to the Slope_S_Africa leading the MCI and the intense reddish zones, where Slope_S_Africa is lagging behind the MCI, are quite neat, serving as an indication of lead-lags regime switching. Third, as for the Level_S_Africa phase-difference heatmap in Fig 5, in the Slope_S_Africa phase difference panel we also observe the red belt for the 4-to-6-weeks frequency band spreads along the whole observation period, meaning that the MCI is leading changes in the level component of the term structure of the South African sovereign debt. Fourth, the overall pattern of the level and slope phase difference heatmap are rather similar, which makes these yield factors not attractive for designing cross-factor level-spread hedge strategies.

These findings seem potentially useful for investment communities, aspiring to thoroughly assess relative attractive ness of investing along the South African sovereign yield curve, as its latent slope factor clearly possesses appealing hedge features, whose hedge-strategy potential for the whole range of investment horizons, remains present through the global crises, as we have evidenced based on the Covid-19 meltdown.

The overall MCI-Curvature_S_Africa patterns exhibited in the two last panels of Fig 5 are very similar to those of the first two panels of this figure. Hence, the corresponding conclusions concerning interrelations of the MCI and volatility of the Curvature_S_Africa are to be the same as in the MCI-Level_S_Africa case. Therefore, we evidence that there is no potential for level-curvature cross-factors hedge strategies. We also do not believe that it is reasonable to design hedge strategies based on curvature yield factor, as the same result could be obtained by using the level yield factor with much less mathematical and portfolio management efforts. Then, we do not believe that the curvature parameter of the South African term structure is an eligible candidate for hedge strategies design due to similarity of its behavior, along the Covid-19 crisis, to the behavior of the level yield factor, while eventual employment of the curvature would bring about an unjustified mathematical and practical complexity.

## 7. Conclusion

The present research scrutinizes the interrelations of the Media Coverage Index (MCI) and volatility of the three factors,–the level, slope, and the curvature,–for the governmental debt term structures of BRICS from January to October, 2020. Wavelet coherence and pha se-difference methods are employed in our study. The research outputs exhibit preponderantly elevated levels of coherence for the yield-factors´ time series and the MCI. The dominating strong coherence is indicative of the elevated levels of correlation between a systemic crisis, such as the one caused by the ongoing coronavirus outbreak, and volatility of BRICS governmental debt. Such high correlation highlights relevance of thorough portfolio management in the case of investing in EM fixed-income securities. Nonetheless, we also report about regions of weak cohere nce, observed for diverse locatiions in the time-frequency space for the considered time series of the latent yield factors. The regions of weak coherence evidence that the yield-shaping components potentially permit harvesting diversification bene fits and may be used as a possible base for creation of hedge strategies during global catastrophes such as the ongoing pandemic. In addition, we evidence and document various contrasting features in the patterns of coherence subjacent to the yield factors for five considered countries.

In particular, we find that the synchronized in-phase behavior of the MCI and level factor of the term structure of Russian governmental debt occurs when the behavior of the MCI and the slope component of the Russian yield-curve is out-of-phase, and vice versa. This makes the Russian government debt to be the most attractive for designing cross-factor hedge strategies. The less attractive in this sense, is South African debt as the patterns of the level and slope phase-difference heatmap are rather similar. In addition, for all the countries, it is concluded that the curvature factor of their term structures is not suitable for designing hedging strategies. It is so as the diversification attributes of the curvature component are very similar to those of the level factor.

Wrapping-up, the results of this study corroborate employing exposures to governmental debt of the BRICS by investors pursuing diversification by means of cross-factor and cross-country hedge strategies, aimed at mitigation of downside risks. Out findings provide relevant insights for investment professionals and market regulators and evidence a high desirability of further investigation in this domain. Portfolio managers and individual investors may use our outcomes for elaborating cross-country, cross-region and cross-factors fixed-income hedging strategies, remaining workable throughout global crises, as demonstrated by our study of the ongoing global coronavirus outbreak. In their turn, financial institutions, in general, and hedge funds, in particular, may come to employ the results of this study to more accurately delineate precise risk profiles of their EM bond portfolios, which more and more become a basilar pillar of the worldwide contemporaneous finance. In parallel, policymakers may consider our findings while working on financial and monetary policies, aimed at reducing mark et turbulences under such uncertain conditions, as are experienced nowadays. Last but not least, further investigation efforts may well target to extend our research by employing diverse methodological alternatives and assessing possible consequences for portfolio management from inclusion of EM debt instruments into a port folio select ion framework.

This study has utilized a bivariate framework for analyzing the impacts of media coverage on the yield-curve shaping components of the BRICS economics. Future research can employ multivariate models and alternative non-linear models such as NARDL (Nonlinear Autoregressive Distributed Lag) to analyze this relationship.

## Supporting information

S1 Data (RAR)
